# Public knowledge, attitudes, and practices during the first wave of COVID-19 in Indonesia

**DOI:** 10.3389/fpubh.2023.1238371

**Published:** 2023-08-03

**Authors:** Rano K. Sinuraya, Chalisma Wulandari, Riezki Amalia, Irma M. Puspitasari

**Affiliations:** ^1^Unit of Global Health, Department of Health Sciences, University of Groningen/University Medical Center Groningen, Groningen, Netherlands; ^2^Department of Pharmacology and Clinical Pharmacy, Faculty of Pharmacy, Universitas Padjadjaran, Bandung, Indonesia; ^3^Center of Excellence for Pharmaceutical Care Innovation, Universitas Padjadjaran, Bandung, Indonesia

**Keywords:** knowledge, attitudes, behavior, COVID-19, awareness, physical distancing, vaccination, public adherence

## Abstract

**Background:**

The COVID-19 pandemic became a global emergency, and it is vital to understand the knowledge, attitudes, and practices of populations regarding this disease to prevent its spread.

**Objective:**

The study aimed to investigate the knowledge, attitudes, and practices of the Indonesian public toward COVID-19.

**Methods:**

An observational study was conducted in Indonesia between November 2020 and January 2021. The study employed a validated questionnaire with 391 respondents to the survey. A comparative analysis was performed to assess the mean differences in respondents’ characteristics in terms of their knowledge, attitude, and practice scores. Furthermore, regression analysis was used to investigate those factors associated with the good practice of health protocols during the COVID-19 pandemic.

**Results:**

The findings showed significant differences in the average knowledge scores across gender, geographical location, and educational attainment. Furthermore, significant differences in practices were identified in terms of gender and educational attainment. The findings of the study indicate that the participants demonstrated a significant awareness of COVID-19, as evidenced by their high mean knowledge score of 17.83 ± 1.74 out of a possible total score of 22 points. The results indicate that the respondents exhibited a positive attitude toward COVID-19 prevention measures, as evidenced by an attitude score of 26.95 ± 3.14 out of a possible total score of 30 points. Additionally, the mean score for good practice in health protocols aimed at reducing COVID-19 infection was found to be high (4.23 ± 0.96) at 5 points. This suggests that the study participants had already adopted effective measures to comply with the recommended health guidelines. The results of the regression analysis indicated that gender, level of education, and knowledge were statistically significant predictors for adherence to health protocols during the COVID-19 pandemic (*p* < 0.05).

**Conclusion:**

The aforementioned results offer significant perspectives into the populace’s comprehension, disposition, and conduct regarding COVID-19 in Indonesia. These insights could potentially contribute to the formulation of efficacious measures aimed at curtailing transmission of the virus.

## Background

The COVID-19 pandemic has presented unprecedented health challenges worldwide and significantly transformed attendant daily routines globally (1). Indonesia reported its initial confirmed COVID-19 cases on March 2, 2020, and the Indonesian government implemented measures to contain the spread of the pandemic, and the COVID-19 Task Force was instituted by the Indonesian government on March 9, 2020, to direct the nation’s reaction to the pandemic (2, 3). The task force was assigned the job of coordinating measures aimed at somewhat preventing the spread of the virus and minimizing its impact on public health and the economy. The government enforced various measures such as travel restrictions, social distancing protocols, and the closure of schools and non-essential businesses to contain the spread of the virus, and the government intensified testing capacity and healthcare infrastructure development in anticipation of a surge in the number of patients (4–6). Up to this point, the most effective strategy for reducing mortality, morbidity, and COVID-19 virus transmission is vaccination. According to the current data from the Ministry of Health of Indonesia (7), since the rollout of COVID-19 vaccination in early 2021, the coverage for first, second, third, and fourth doses stands at 86, 74, 38, and 1.8%, respectively.

The COVID-19 outbreak prompted swift distribution of information and guidance by public health authorities (8), but misinformation and misunderstandings regarding the disease have been prevalent, resulting in perplexity and possibly detrimental behaviors (9–11). Public attitudes toward COVID-19 played a crucial role in mitigating the transmission of the virus, and these attitudes can impact adherence to public health measures, including social distancing, mask-wearing, and vaccination (12). In general, the attitudes of people may be influenced by various factors, including but not limited to their level of trust in the public health authorities, their perception of risk, and their perception of personal susceptibility (12, 13). Individual behavior is seen as a crucial element in the transmission of COVID-19, alongside knowledge and attitudes (14), and evaluating the public’s adherence to COVID-19 prevention measures is therefore essential in managing the disease’s transmission.

Therefore, accurate knowledge and appropriate preventive measures are essential for controlling the spread of the virus (15), and evaluating the public’s knowledge, attitudes, and practices regarding COVID-19 is imperative for delivering effective public health interventions and targeted communication strategies within the context of the evolving pandemic (14–17). This study aims to evaluate the COVID-19-related knowledge, attitudes, and practices of the public to identify knowledge gaps and intervention requirements, and the research will employ a comprehensive survey approach to gather information on COVID-19-related knowledge, attitudes, and practices from a representative sample of the population. This study’s findings will help the current endeavors to manage the pandemic and safeguard public health by pinpointing specific regions where focused interventions and strategies for communication can be executed.

## Materials and methods

A cross-sectional study of the Indonesian population was carried out between November 2020 and January 2021. Data were collected through a self-reported online questionnaire, deployed due to social distancing measures, restricted movements, and lockdowns. The survey was distributed to respondents through email and WhatsApp groups. The study’s participants were at least 18 years old, had electronic devices and internet access, and provided their informed consent to take part in the study. The cross-sectional survey’s sample size was determined using a conventional normal variation of type 1 error at 5%, an anticipated proportion of participants at 50%, and an absolute error at 5%. The researchers determined that the appropriate sample size was 384 participants; however, this study could actually recruit up to 391 respondents. Participants were informed that the results of this voluntary study would be used for research only. The study received approval from the Health Research Ethics Committee of Universitas Padjadjaran, Indonesia (registration number: 1011/UN.6.KEP/EC/2020) and was conducted in compliance with the Helsinki Declaration.

The knowledge, attitudes, and practices questionnaire, along with the scoring system, were adapted and professionally translated into Indonesian based on a prior study (18). The survey comprised four sections: respondent background, knowledge assessment (22 items), attitude assessment (6 items), and practice assessment (5 items). For the knowledge section, a score of 1 was assigned to correct responses, while incorrect responses received a score of 0. Attitude responses were evaluated using a Likert scale consisting of five options: strongly disagree (scored 1), disagree (scored 2), neutral (scored 3), agree (scored 4), and strongly agree (scored 5). In the practice section, respondents were assigned a score of 1 for demonstrating good practices and a score of 0 for responses indicating bad practices.

Results data were saved in a secure digital repository. A *t*-test was used to examine the variations between knowledge, attitudes, and practice in terms of the age, gender, and region of the respondents. Meanwhile, an ANOVA test was used to evaluate the level of education reached. Linear regression analysis was performed to determine the variables that were associated with good practice during the pandemic. The statistical analyses were performed with Statistical Package for Social Sciences (SPSS) 27 software (SPSS Inc., Chicago, IL, United States).

A face validation was conducted by two investigators (RKS and IMP), followed by a reliability test that involved 30 respondents. The questionnaire’s Cronbach’s alpha scores for knowledge, attitude, and practice were considered acceptable with values of 0.82, 0.84, and 0.84, respectively. Therefore, it was determined by testing that the questionnaire was valid and reliable for use in this study (19, 20). The general characteristics were presented using descriptive statistics. A linear multiple regression analysis was performed to determine the variables that impact successful practice in terms of the pandemic. Autocorrelation was deemed not present based on the use of a Durbin–Watson coefficient of 2. The presence of multicollinearity was evaluated through the use of the tolerance level and the variation inflation factor, with established thresholds of 0.53–0.98 and 1.02–1.85, respectively. A regression analysis was performed and resulted in a value of p of less than 0.001. The mean residual standard was found to be 0, and the range was below 3, suggesting there were no outliers. Based on the criteria of normality, residual, multicollinearity, regression test, and independent error, it was determined that the linear regression model was appropriate.

## Results

Three hundred and ninety-one respondents completed the questionnaire. The majority of the respondents were recent graduates from college or bachelor’s degree programs, resulting in a higher proportion of respondents aged ≤ 22. Of the respondents, 19.7% were male and 80.3% were female, with the percentage of individuals above 22 years old being 63.3% for males and 31.8% for females. The majority of the respondents were from the Java Islands (80.8%), and 32 and 48% of the respondents were graduates of high school and undergraduate school, respectively. Meanwhile, 19.7% of the participants were graduates of vocational school and graduate school. The mean COVID-19 knowledge score was 17.83 (SD = 1.74, range: 7–21). The mean attitude score for COVID-19 was 26.95 (SD = 3.14, range: 6–30), indicating positive attitudes towards implementing health protocols. The mean score for practices in terms of COVID-19 was 4.23 (SD = 0.96, range: 0–5), indicating good practices (Table 1).

**Table 1:** Characteristics of respondents (*N* = 391).

Variables	*N* (%)	Mean	SD	Min.	Max.
Age (years)		24.5		19	45
≤22	242 (61.9)				
>22	149 (38.1)				
Gender					
Male	77 (19.7)				
Female	314 (80.3)				
Region					
Non-Java	75 (19.2)				
Java	316 (80.8)				
Education level attained					
Senior high school	126 (32.2)				
Vocational school	28 (7.2)				
Undergraduate	188 (48.1)				
Graduate school	49 (12.5)				
Knowledge		17.83	1.74	7	21
Attitude		26.95	3.14	6	30
Practice		4.23	0.96	0	5

SD (standard deviation), min (minimum), max (maximum).

Two questions related to COVID-19 main symptoms and the susceptibility of pregnant women to infection demonstrated a low rate of correct responses. Most respondents demonstrated knowledge of COVID-19 transmission through particles or droplets and the importance of maintaining hygiene by washing hands regularly and avoiding touching the face. The majority of respondents demonstrated a good understanding of conducting self-quarantine, as indicated in Table 2. Interestingly, up to 40% of the respondents provided incorrect responses regarding the use of medical face masks. This discrepancy may have occurred due to the short implementation period of the previous policy, which recommended that medical face masks be used exclusively by healthcare professionals in healthcare facilities.

**Table 2:** Participants’ responses to the questions on COVID-19 knowledge.

Items	*N* (%)	
	Correct response	Incorrect response
COVID-19 spreads within 2 m	318 (81.3)	73 (18.7)
COVID-19 spreads through coughing or sneezing	388 (99.2)	3 (0.8)
Touching objects with the virus can lead to infection	385 (98.5)	6 (1.5)
Eating wild animals can cause COVID-19	343 (87.7)	48 (12.3)
No transmission if COVID-19-positive individuals lack a fever	340 (87)	51 (13)
Main symptoms of COVID-19: fever, fatigue, cough, muscle pain, shortness of breath	384 (98.2)	7 (1.8)
Unlike common cold, individuals infected with SARS-CoV-2 rarely experience nasal congestion, runny nose, and sneezing.	94 (24)	297 (76)
Antibiotics are effective for COVID-19	258 (66)	133 (34)
No cure, but symptom-based treatment helps to heal	371 (94.9)	20 (5.1)
Higher risk for adults and those with chronic illnesses	380 (97.2)	11 (2.8)
Only chronically ill adults are likely to have a severe infection	285 (72.9)	106 (27.1)
Pregnant women are more vulnerable to COVID-19	46 (11.8)	345 (88.2)
Children have a lower risk of contracting COVID-19	111 (28.4)	280 (71.6)
Children/teens need not follow health protocols	379 (96.9)	12 (3.1)
Wash or sanitize your hands after being in public places	378 (96.7)	13 (3.3)
Avoid touching your face without washing your hands	391 (100)	0 (0)
The public can wear general medical masks to prevent infection	231 (59.1)	160 (40.9)
Masks should be used if infected or caring for the infected	363 (92.8)	28 (7.2)
Healthy eating boosts immunity to COVID-19	378 (96.7)	13 (3.3)
Isolation/treatment reduces COVID-19 spread	379 (96.9)	12 (3.1)
Quarantine for close contacts of COVID-19 patients for 14 days	389 (99.5)	2 (0.5)
Avoid crowds and limit your use of public transportation to prevent transmission	383 (98.0)	8 (2.0)

Table 3 shows the respondents’ attitudes toward COVID-19 infection. Many participants expressed their willingness to adhere to health protocols, including social distancing and personal hygiene. Although most of the respondents answered positively, some expressed uncertainty about the effectiveness of the health protocols implemented by the Indonesian government in controlling the infection, as indicated in Table 3. This is understandable since the infection and mortality rates were very high in the first wave of infection.

**Table 3:** Participants’ responses to the questions on attitudes toward COVID-19.

Items	*N* (%)				
	Strongly disagree	Disagree	Neutral	Agree	Strongly agree
Social distancing is crucial to prevent the spread of COVID-19	4 (1)	1 (0.3)	4 (1)	37 (9.5)	345 (88.2)
Regular handwashing after going outside is vital for protection against COVID-19	4 (1)	0 (0)	2 (0.5)	25 (6.4)	360 (92.1)
To protect myself from COVID-19 exposure, I should stay home when I’m sick, except for medical care	5 (1.3)	7 (1.8)	16 (4.1)	75 (19.2)	288 (73.7)
COVID-19 will be controlled eventually	5 (1.3)	13 (3.3)	75 (19.2)	109 (27.9)	189 (48.3)
Government health protocols can reduce COVID-19 cases in Indonesia	7 (1.8)	22 (5.6)	90 (23)	132 (33.8)	140 (35.8)
Community compliance with health protocols prevents the spread of COVID-19	3 (0.8)	8 (2)	33 (8.4)	106 (27.1)	241 (61.6)

This survey was conducted 8 months after the first confirmed case of COVID-19 in Indonesia. As shown in Table 4, all respondents reported implementing social distancing and adhering to health protocols, such as avoiding large gatherings, maintaining personal hygiene, and not shaking hands.

**Table 4:** Participants’ responses to the questions on practices regarding COVID-19.

Items	*N* (%)	
	Yes	No
Attended a crowded event recently	85 (21.7)	306 (78.3)
Visited crowded places recently	135 (34.5)	256 (65.5)
Avoiding handshakes lately	349 (89.3)	42 (10.7)
Practicing social distancing	372 (95.1)	19 (4.9)
Washing hands regularly for at least 40 s, especially after being in public or coughing/sneezing	373 (95.4)	18 (4.6)

Table 5 presents the variations in knowledge, attitudes, and practices of participants across different characteristics. The analysis revealed significant differences in knowledge among respondents based on their gender, region, and educational background. Specifically, females (value of *p* 0.013), those residing in Java (value of *p* 0.011), and college or university graduates (value of *p* 0.008) exhibited a higher level of COVID-19 knowledge. Additionally, the study found that good practices in maintaining health protocols were observed more frequently among female respondents generally (value of *p* 0.002) and specifically college or university graduates (value of *p* 0.003).

**Table 5:** Comparison of participants’ characteristics and mean scores of knowledge, attitudes, and practices.

Variables	*N*	Knowledge			Attitude			Practice		
		Mean	SD	*p*-value	Mean	SD	*p*-value	Mean	SD	*p*-value
*Age (years)* *^a^*										
≤22	242 (61.9)	17.94	1.62	0.144	27.03	2.68	0.564	4.28	0.91	0.173
>22	149 (38.1)	17.66	1.92		26.82	3.78		4.14	1.04	
*Gender* *^a^*										
Male	77 (19.7)	17.24	2.41	0.013*	26.53	3.86	0.261	3.85	1.18	0.002*
Female	314 (80.3)	17.98	1.51		27.06	2.93		4.32	0.88	
*Region* *^a^*										
Non-Java	75 (19.2)	17.18	2.58	0.011*	26.65	3.68	0.411	4.12	1.02	0.275
Java	316 (80.8)	17.99	1.44		27.03	3.00		4.26	0.95	
*Education level attained* *^b^*										
High school	126 (32.2)	17.59	2.01	0.008*	27.02	2.36	0.409	4.09	1.03	0.003*
Vocational	28 (7.2)	17.07	2.35		26.17	3.58		3.75	1.35	
Undergraduate	188 (48.1)	18.03	1.49		26.91	3.65		4.36	0.83	
Graduate school	49 (12.5)	18.16	1.29		27.42	2.39		4.36	0.86	

* Significant *p* < 0.05.

a *t*-test.

b ANOVA test.

SD (standard deviation).

To identify variables associated with good practices in reducing infection and mortality rates, this study conducted linear multiple regression analysis. Six variables, namely, age, gender, education, region, knowledge, and attitudes, were examined, and the explanatory power of these factors in influencing good practices was found to be 7.5% (*F* = 4.907, value of *p* < 0.001; Table 6). Among these variables, gender (*β* = 0.405), undergraduate (*β* = 0.312), and graduate school (*β* = 0.413) were identified as the most influential factors for predicting good practice in adhering to health protocols for COVID-19 (see Table 6).

**Table 6:** Factors associated with good practice in minimizing COVID-19 infections.

Variables	*β*	SE	*t*	*p*-value	95%CI
Age (Ref: ≤ 22)	−0.149	0.126	−1.178	0.240	−0.397–0.100
Gender (Ref: Male)	0.405	0.125	3.246	0.001^*^	0.160–0.651
Region (Ref: Java)	0.036	0.125	0.286	0.775	−0.210–0.282
Education level attained (Ref: High school)					
Vocational school	−0.146	0.216	−0.674	0.500	–0.570–0.279
Undergraduate	0.312	0.115	2.721	0.007^*^	0.086–0.539
Graduate school	0.413	0.193	2.227	0.027^*^	0.050–0.811
Knowledge	0.057	0.028	2.021	0.044^*^	0.002–0.113
Attitude	0.012	0.015	0.790	0.430	−0.018–0.042
Constant	2.398	0.616	3.896	<0.001	1.188–3.609

* Significant *p* < 0.05.

*R* = 0.307, Adjusted *R*^2^ = 0.075, *F* = 4.953, *p* < 0.001.

SE (standard error).

## Discussion

COVID-19 is a recently identified infectious disease that presents a substantial risk to the well-being of the population (1). In light of the significant risks posed by COVID-19 and current vaccine hesitancy, preventive measures are crucial in mitigating infection rates and managing the transmission of the disease (1, 21). The findings from the study provide a significant contribution to an understanding of the Indonesian population’s knowledge, attitudes, and behaviors regarding COVID-19. Understanding and assessing these variables is crucial in developing successful approaches to manage the transmission of the virus and reduce the impacts of the pandemic.

The results of our study suggest that the majority of participants possess a satisfactory level of understanding regarding COVID-19. The knowledge, attitudes, and practices questionnaire was administered to the study participants, and the results indicated a mean score of 17.83 (81.05%), which means that people are quite knowledgeable about COVID-19. Even though this investigation was carried out 8 months after the initial case was detected in Indonesia, it is evident that the populace exhibits awareness and adequate comprehension of this disease. Similar to a previous study conducted in Albania (22), our findings revealed that the participants displayed a high level of precision in responding to knowledge-based inquiries, which was an expected outcome. This observed phenomenon can potentially be attributed to the demographic characteristics of the participants. Specifically, a significant proportion (67.8%) possessed a tertiary education qualification or higher, and all participants were below the age of 50. The potential cause of this outcome may also be attributed to the dissemination of the survey during the pandemic, because during the initial wave of COVID-19 in Indonesia, the government worked closely with local health authorities to distribute information about the virus widely across various media channels and individuals may have acquired knowledge and awareness of the disease as a result of this (8).

The majority of the study’s participants demonstrated knowledge of the clinical symptoms associated with COVID-19 (98.2%), high-risk individuals (97.2%), and treatment based on symptoms (94.9%). Moreover, the majority of respondents, specifically, over 80%, demonstrated knowledge regarding the transmission of COVID-19 through proximate, person-to-person contact. However, it was evident that a significant proportion of the current population (59%) had a limited understanding of who should wear a mask and when as a preventive measure against infection. As per the Indonesian COVID-19 task force’s reports (23), until the beginning of 2022, a considerable proportion of individuals continued to disregard health protocols, such as wearing masks and maintaining social distancing.

The government of Indonesia has taken various country-wide measures aimed at mitigating the spread of COVID-19. These endeavors consist of initiatives aimed at raising public consciousness regarding the virus and advocating for responsible conduct (8, 24). Comprehensive health protocols and guidelines have been established, encompassing various aspects such as mask-wearing, adherence to social distancing measures, and the implementation of hygiene practices. Testing, tracing, and isolation tactics have been used to detect cases of infection, oversee individuals who have been in contact with those impacted, and hinder the spread of the disease (5, 8). The prioritization of high-risk groups has been a key focus of vaccination campaigns, while measures such as travel restrictions and border controls have been implemented to reduce the transmission of the virus from external sources (24).

The study identified gender, region, and educational level as significant predictors of participant knowledge. This discovery is corroborated by prior research that has demonstrated that female individuals who have higher levels of education exhibit greater awareness regarding emergent infectious illness (25, 26). The results of our investigation emphasize the significance of giving priority to the use of mass media to aim at particular demographic groups, including males in general, and those individuals with lower educational attainment. By implementing targeted interventions and customizing educational programs, public health officials can enhance the general populace’s comprehension of the pandemic effectively (27).

In terms of attitudes, the findings of the study indicate that the individuals canvassed demonstrate a favorable and hopeful outlook toward the COVID-19 outbreak. A significant percentage of respondents, specifically 76.2%, held the view that it is possible to implement efficacious measures to manage the transmission of the virus. Additionally, the research revealed that a majority, 69.6% of participants, exhibited trust in the capacity of the Indonesian government to handle the COVID-19 outbreak. This indicates a noteworthy degree of confidence in the government’s endeavors and tactics to manage the crisis proficiently. The fact that most participants exhibit a favorable perception of the government’s management is significant, as it has the potential to enhance adherence to public health guidelines and policies. This finding underscores the significance of clear and open communication, efficient administration, and salient anticipatory actions in fostering public reliance and assurance in difficult circumstances. The findings of a cross-national survey by Han et al. (28) for example indicated a significant correlation between an increased level of confidence in governmental institutions concerning the management of COVID-19 and the increased uptake of healthy and prosocial behaviors. Trust was found to be impacted positively by various factors, such as the perception of the government’s organizational efficiency, its proficiency in disseminating clear and accurate information regarding COVID-19, and a perceived sense of equity (29).

The results of our study emphasize a significant association between the participants’ comprehensive understanding of COVID-19 and their subsequent compliance with precautionary measures during the pandemic. The data suggest that Indonesian residents exhibit a significant level of caution in their conduct, which is indicative of their awareness of the potential hazards linked with the virus. It is noteworthy that a considerable majority, comprising around 78% of the respondents, opted to refrain from participating in social events. This is a responsible course of action aimed at mitigating the likelihood of transmission. Additionally, a majority, 66% of the participants, implemented preventive measures by refraining from visiting crowded places, thereby demonstrating their dedication to mitigating the risk of contracting the virus.

An additional noteworthy finding from the research is that a significant proportion of the participants, specifically 89%, abstained from shaking hands, a customary gesture that has the potential to expedite the transmission of the virus. This discovery showcases a notable degree of adherence to the prescribed hygiene protocols, which indicates that the participants understand the importance of refraining from physical contact as a preventive measure against transmission. The findings of our research indicate that the population of Indonesia exhibits a significant level of awareness regarding COVID-19, as demonstrated by the observance of precautionary measures in the ongoing health crisis.

This study, which was carried out in the months following the occurrence of the first recorded case in Indonesia, provided significant findings regarding the efficacy of health authorities’ initiatives or interventions in raising public consciousness during the pandemic. The implications of the study’s findings are promising in terms of providing information for proactive measures in potential future occurrences. It is however important to recognize specific constraints that need to be considered when interpreting the findings of this investigation.

A significant limitation of this study is its reliance on self-reported data, which may introduce reporting bias, particularly when using the online survey method. To address this issue, future studies could explore the use of administrative data, which would provide a more objective and reliable evaluation of the variables under examination. We did not include the working status in the survey because during the pandemic, many private sectors were closed, resulting in job losses and significant economic impact. Additionally, it would be beneficial to include personal attributes in specific variables, such as individuals’ attitudes toward and expectations of the government, as well as their hygiene practices, across respondents of all age groups. Despite these constraints, the investigation yields valuable insights into the awareness and attitudes of individuals during the initial phases of the pandemic. This study serves as a foundation for future research and guides public health interventions.

## Conclusion

The findings of this study reveal that the population exhibited good knowledge of COVID-19 and demonstrated a strong awareness in implementing health protocols. Specifically, the results indicate that the people of Indonesia, particularly women, have a commendable level of knowledge and adhere to recommended practices related to the pandemic. Notably, individuals residing on Java Island, with higher levels of education, exhibit a comprehensive understanding of COVID-19. It is crucial to note that gaining a comprehensive understanding of the disease is considered the initial step towards implementing effective healthcare educational initiatives.

## Data availability statement

The datasets for this article are not publicly available due to concerns regarding participant/patient anonymity. Requests to access the datasets should be directed to the corresponding author.

## Ethics statement

The studies involving human participants were reviewed and approved by Health Research Ethics Committee of Universitas Padjadjaran, Indonesia (registration number: 1011/UN.6.KEP/EC/2020). The patients/participants provided their written informed consent to participate in this study.

## Author contributions

RKS, CW, RA, and IMP played a significant role in the conception and design of the study, as well as the collection, analysis, and interpretation of the data. RKS and CW prepared the initial draft of the manuscript, while subsequent revisions were made by RA and IMP. All authors contributed to the article and approved the submitted version.

## Funding

This study was funded by Universitas Padjadjaran through a research grant for RA (Grant number: 1397/UN6.3.1/PM/2020).

## Conflict of interest

The authors declare that the research was conducted in the absence of any commercial or financial relationships that could be construed as a potential conflict of interest.

## Publisher’s note

All claims expressed in this article are solely those of the authors and do not necessarily represent those of their affiliated organizations, or those of the publisher, the editors and the reviewers. Any product that may be evaluated in this article, or claim that may be made by its manufacturer, is not guaranteed or endorsed by the publisher.
